# Diffusion‐Weighted Magnetic Resonance Imaging: A Diagnostic Tool for Auditory (Axonal) Neuropathy

**DOI:** 10.1111/ene.70083

**Published:** 2025-02-11

**Authors:** Julien Zanin, Gary Rance

**Affiliations:** ^1^ Department of Audiology and Speech Pathology The University of Melbourne Parkville Melbourne Australia

**Keywords:** auditory neuropathy, axonopathy, diffusion‐weighted MRI, hearing loss, neuroimaging

## Abstract

**Background:**

Axonal neuropathies are disorders that impair neural transmission, leading to substantial sensory deficits. In the auditory system, axonal degeneration can disrupt auditory processing, causing significant hearing difficulties. Understanding the extent of axonal degeneration and its impact on auditory function is crucial for improving diagnosis and management. This study aims to quantify axonal degeneration in the VIIIth nerve using diffusion‐weighted MRI and to correlate these findings with auditory function.

**Methods:**

Fifty‐two children and adults participated. A total of, 27 with normal hearing, 7 with cochlear hearing loss and 18 with auditory neuropathy (AN). Hearing thresholds and dMRI data was collected for all participants and the VIIIth nerve was evaluated using the fixel‐based analysis metric of Apparent Fibre Density (AFD).

**Results:**

AFD was significantly lower in participants with AN compared to participants with normal hearing and cochlear hearing loss (*p* < 0.05). 9/18 participants with AN exhibited AFD values ≥ 2 standard deviations below the normal range. Additionally, AFD was strongly correlated with hearing thresholds in participants with no evidence of cochlear dysfunction (*r* = −0.776, *p* < 0.001), suggesting reduced auditory nerve fibre density is associated with impaired sound detection.

**Conclusions:**

dMRI‐derived AFD is a sensitive marker for axonal degeneration in the VIIIth nerve. This study provides the first in vivo evidence linking VIIIth nerve microstructure with hearing thresholds, highlighting the potential of dMRI in diagnosing and monitoring AN. The findings suggest that dMRI could be a valuable tool in clinical settings for assessing auditory nerve health and guiding treatment strategies for individuals with AN.

## 1 Introduction

Axons are the long, thin fibres of neurons that form the wiring of the nervous system. During the ageing process, around 40% of these fibres are lost [1], including those of the auditory and visual systems [2, 3]. They are also vulnerable to trauma and can undergo premature degeneration due to various acquired or inherited conditions.

Axonal neuropathies (also known as axonopathies) are a diverse group of disorders that impair the nervous system's ability to transmit electrical impulses efficiently. These conditions are often classified based on the diameter of the affected axons: large fibre neuropathies primarily impact motor and some sensory modalities (including touch), whereas small fibre neuropathies affect other sensory modalities and autonomic functions [4]. Within the auditory system, myelinated fibres, with their relatively small diameters (~5–6 μm) [5], are especially susceptible to damage, which can severely disrupt the neural representation of auditory stimuli [6].

Axonopathies directly impact the magnitude of the compound action potential, which is correlated with the degree of reduction in synchronised neuronal activity due to loss of nerve fibres or secondary demyelination. The resulting disruption in firing patterns, in turn, affects the representation of temporal (timing) information in the central auditory pathways. As a result, speech understanding [7, 8] which is dependent on the precise representation of temporal cues, may be adversely affected. Furthermore, temporal accuracy is crucial for binaural processing, and hence, listening/communication in background noise. Functional hearing deficits involving speech understanding and everyday communication are commonly reported in patients with axonal conditions such as Friedreich ataxia (FRDA), Charcot–Marie–Tooth disease (type 2; CMT2) and riboflavin transporter deficiency (RTD) [7, 9, 10, 11]. Moreover, these auditory deficits can often occur in the absence of significantly elevated hearing thresholds [12].

The pathophysiological mechanism underpinning auditory deficits in axonopathies differs from cochlear hearing loss, which is primarily caused by damage and degeneration of the sensory hair cells of the cochlea, particularly the outer hair cells [13, 14]. Outer hair cells play a vital role in the ability to hear soft sounds, and therefore, a diagnosis of cochlear hearing loss is dependent on elevated hearing thresholds and reduced speech discrimination. Neural synchrony is largely unaffected, and once audibility has been accounted for (e.g., through amplification), temporal processing remains relatively intact [15]. In contrast, individuals with axonopathy, which affects the neural pathways rather than the sensory cells, can present with normal or near‐normal hearing thresholds but significantly reduced temporal processing ability and substantial difficulties in understanding speech, especially in noisy environments. These distinct clinical features emphasise the importance of tailored diagnostic tools and approaches to differentiate cochlear hearing loss from neural auditory deficits. Some degree of neural degeneration within peripheral axons, however, has been shown to occur in cochlear hearing loss associated with aging and excessive noise exposure [6, 16, 17].

Our understanding of axonal degeneration within the auditory system comes mainly from post‐mortem histological studies as there are currently no diagnostic assessments capable of quantifying the extent of neuronal loss [12]. Although electrophysiological assessment, such as the auditory brainstem response (ABR), can help to identify individuals exhibiting auditory neural abnormality, these assessments do not provide information about the extent of disruption or underlying pathology. Similarly, structural MRI may identify absent or hypoplastic auditory nerves, but findings are not always definitive—especially in cases where the cochlear nerve is thin or has undergone degenerative changes [18, 19].

Diffusion‐weighted MRI (dMRI) is a non‐invasive imaging acquisition strategy, available on clinical MRI scanners, that has transformed the ability to study white matter (WM) architecture in vivo. Since the water‐molecule diffusion characteristics differ substantially between WM, grey matter and cerebrospinal fluid, mathematical models can use this information to identify the location, orientation, and organisation of the fibre pathways [20]. Moreover, quantitative metrics of WM microstructure can be derived, providing an indication of axonal density within specific WM bundles. These techniques have previously been used to assess and quantify WM abnormality and degeneration within the VIIIth cranial nerve and auditory brainstem in patients with auditory neuropathies [18, 21].

This study leveraged the capabilities of dMRI to investigate microstructural changes associated with axonal neuropathies in the auditory system, quantifying the degree of VIIIth nerve degeneration/damage and correlating these with functional hearing deficits.

## 2 Materials and Methods

### 2.1 Participants

A total of 52 children and adults participated in the study, including 27 with normal hearing, 7 with cochlear hearing loss, and 18 with auditory neuropathy (AN). Participants with AN were recruited through the People's Liberation Army General Hospital (PLAGH; Beijing), and the University of Melbourne Neuroaudiology Clinic. Normal hearing participants were recruited through friends and colleagues of the authors, while the participants with cochlear hearing loss were recruited through the University of Melbourne Audiology Clinic and the Royal Victorian Eye and Ear Hospital.

**FIGURE 1:**
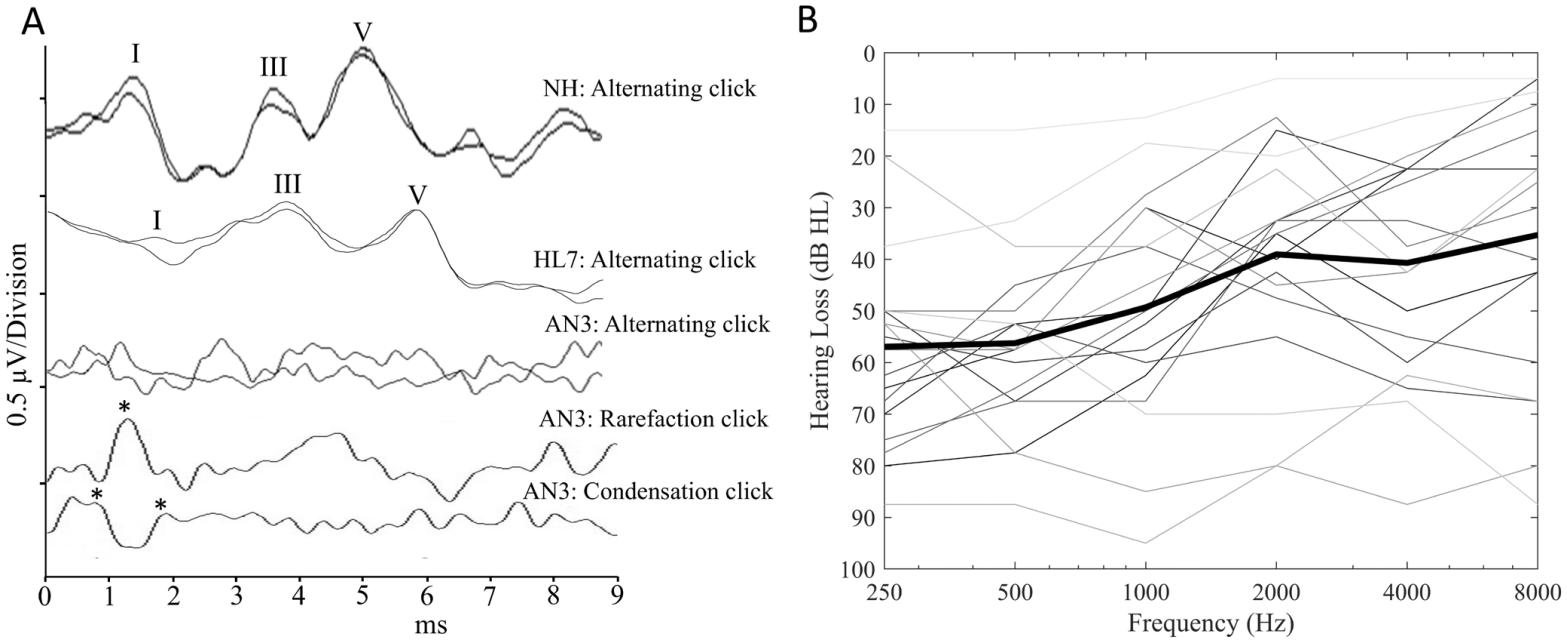
(A) Auditory Brainstem Response recordings to 90dBnHL acoustic click stimuli. The top trace was recorded from a normal hearing (NH) control participant and is a typical result obtained from this cohort. The second trace shows the result obtained from participant #7 with hearing loss (HL7) from their better hearing ear. The lower traces were recorded from participant #3 and are representative of the type of results obtained from the participants with auditory neuropathy (AN). In these cases, the main peaks (Wave I, III and V) are absent, however, rarefaction and condensation polarity stimuli can be used to identify the cochlear microphonic (CM) potential (positive peaks of the CM are shown by the asterisks). (B) Pure tone audiometric thresholds averaged across both ears for the participants with auditory neuropathy. Bold black line represents the average of all the individual hearing thresholds shown.

The aetiology of the AN‐phenotype for each of the AN participants is shown in Table 1. Participants were classified as having AN if they showed an absent or disordered ABR and evidence of pre‐neural cochlear function, including the presence of either distortion product otoacoustic emissions (DPOAEs) or cochlear microphonics (Figure 1A). Participants were determined to have normal auditory function if they exhibited extant ABRs and hearing thresholds of ≤ 20 dBHL from 250 to 8000 Hz. Individuals with AN ranged in age from 9 to 65 years (mean: 29.3; SD: 15.9 years). For the cochlear hearing loss group, ages ranged from 10 to 67 years (mean: 32.9; SD: 24.2 years) and participants with normal hearing were aged 8–43 years (mean: 21.0; SD: 8.8 years). Demographic and clinical details are provided for the participants with AN and cochlear hearing loss in Table 1. Pure tone audiometric thresholds averaged across both ears for the AN participants are shown in Figure 1B. Structural T1 and T2‐weighted MR images for all participants were reviewed by a radiologist and/or an ear, nose, and throat specialist. These showed no direct evidence of hypoplastic auditory nerves.

**TABLE 1:** Demographic and audiometric details for the auditory neuropathy (AN) and cochlear hearing loss (HL) participants.

Subject	Age (years)	Duration (years)^a^	Gender	Aetiology^b^	Audiometry				
					4FAHL (L/R)	HFAHL^c^ (L/R)	OAEs (L/R)	ABR	CM (L/R)
AN1	24	15	M	AUNX1	50/62.5	41.7/43.3	+/+	Absent	+/+
AN2	18	7	M	AUNX1	42.5/32.5	28.3/16.7	+/+	Absent	+/+
AN3	23	9	M	AUNX1	33.8/36.3	20/20	+/+	Absent	+/+
AN4	18	4	M	AUNX1	46.3/41.3	20/20	+/+	Absent	+/+
AN5	25	14	M	AUNX1	62.5/47.5	66.7/30	+/+	Absent	+/+
AN6	56	40	M	AUNX1	57.5/58.8	68.3/56.7	+/+	Absent	+/+
AN7	40	29	M	AUNX1	55/37.5	71.7/36.7	+/+	Absent	+/+
AN8	47	33	M	AUNX1	48.8/51.3	36.7/33.3	+/+	Absent	+/+
AN9	39	22	M	AUNX1	37.5/50.0	26.7/23.3	+/+	Absent	+/+
AN10	43	27	M	AUNX1	33.8/30.0	26.7/26.7	−/−	Absent	+/+
AN11	42	24	M	AUNX1	48.8/38.8	48.3/26.7	+/+	Absent	+/+
AN12	18	5	M	AUNX1	38.8/38.8	25/16.7	+/+	Absent	+/+
AN13	21	16	F	RTD2	76.7/77.5	81.7/83.3	+/+	Absent	+/+
AN14	18	3	F	RTD2	73.3/86.7	65/75	+/+	Absent	+/+
AN15	16	8	F	RTD2	23.3/37.5	21.7/36.7	+/+	Absent	+/+
AN16	65	0	F	MELAS	67.5/71.0	71.7/78.3	−/−	Absent	+/+
AN17	11	1	F	FRDA	20/21.3	13.3/13.3	+/+	Abnormal	+/+
AN18	9	3	M	CMT2C	11.7/7.5	5/5	+/+	Abnormal	+/+
HL1	11	11	M	FH	62.5/54.4	81.7/61.7	−/−	Present	+/+
HL2	18	18	F	FH	38.7/42.5	43.3/81.7	−/−	Present	+/+
HL3	16	16	F	Nil	74.8/75	83.3/86.7	−/−	Absent	−/−
HL4	13	13	M	Nil	75/78.8	90/93.3	−/−	Absent	−/−
HL5	58	7	M	NIHL	38.7/42.5	40/55	−/−	Present	+/+
HL6	49	?	F	Nil	83.8/97.5	96.7/105	−/−	Absent	−/−
HL7	67	11	M	NIHL + SSNHL	33.8/> 110	61.7/> 110	−/−	Present	+/−

Abbreviations: 4FAHL, 4‐frequency average hearing loss; ABR, auditory brainstem response; AUNX1, X‐linked auditory neuropathy; CM, cochlear microphonic; CMT2C, Charcot–Marie‐Tooth type 2C; FH, family history; FRDA, Friedreich ataxia; L, left ear; NIHL, noise induced hearing loss; OAEs, otoacoustic emissions; R, right ear; RTD, riboflavin transporter deficiency type 2; SSNHL, sudden sensorineural hearing loss.

^a^ Duration of diagnosis/auditory symptoms.

^b^ Aetiology was determined through either genetic testing, neurological examination, or a comprehensive history.

^c^ High‐frequency average hearing loss (HFAHL) includes thresholds at 2‐, 4‐ and 8 kHz.

### 2.2 Pure Tone Audiometry

Hearing thresholds were assessed at octave frequencies from 250 Hz to 8 kHz in either a sound‐attenuating test booth or a quiet environment with noise levels ≤ 40 dBA. A four‐frequency average hearing level (4FAHL), comprising 0.5, 1, 2 and 4 kHz test frequencies across both ears, was calculated and used in further analyses.

### 2.3 Otoacoustic Emissions

Distortion product otoacoustic emissions were recorded at 500, 1, 2 and 4 kHz. Stimulus tones f1 and f2 were presented at 65‐ and 55‐dB SPL, respectively, with an f2/f1 ratio of 1.21. A response amplitude of ≥ 6 dB for three consecutive frequencies was required to consider an otoacoustic emission present.

### 2.4 Auditory Brainstem Response

Auditory brainstem responses were recorded to alternating polarity 100 μs clicks at 90–100 dB nHL. A stimulus presentation rate of 8 Hz was used, and two waveforms with 2000 post‐stimulus samples were obtained per ear. Potentials were recorded using silver/silver chloride surface electrodes. Two experienced audiologists (JZ/GR) analysed the waveforms to identify ABR waves I, III and V based on repeatability and latency. In cases of absent ABR, waveforms were analysed for the presence of a cochlear microphonic as per Rance et al. (1999) [22].

### 2.5 Magnetic Resonance Imaging Data

Three different 3‐Tesla Siemens scanners were used in the acquisition of the imaging data. The MRI data collected in China was acquired using a Magnetom Skyra system, whereas in Australia a Magnetom Skyra and a Prisma system were employed. Diffusion‐weighted MRI was performed using a twice‐refocused spin‐echo, echo‐planar imaging sequence (repetition/echo time = 8400/110 ms, 2.5 mm isotropic voxels, field of view = 240 × 240 mm, matrix size = 96 × 96, acceleration factor = 2). A total of 64 diffusion‐weighted images (*b* = 3000 s/mm^2^) and 8 non‐diffusion‐weighted images (*b* = 0 s/mm^2^) were acquired within ~10 min. High‐resolution T2‐weighted images (voxel size = 0.5 × 0.5 × 2.0 mm^3^, repetition/echo time = 4750/101 ms, flip angle = 150°, slice thickness = 2 mm) were also acquired.

### 2.6 Processing of Diffusion‐Weighted MRI Data

The dMRI data was pre‐processed prior to undergoing analysis. This included denoising [23], Gibbs‐ringing artefact removal [24] and eddy‐current and motion correction [25]. Spatial resolution was upsampled to a resolution of 1.3mm^3^ voxel size using cubic b‐spline interpolation. Single‐shell 3‐tissue constrained spherical deconvolution (performed using mrtrix3tissue v5.2.9; https://3tissue.github.io/) [26] was used to compute fibre orientation distribution functions (ODFs) based on group‐averaged response functions for white matter, grey matter and CSF [27]. The white matter ODF images for each individual were used to create a study‐specific, unbiased ODF population template using linear and nonlinear registration techniques [28]. Whole‐brain probabilistic tractography produced 20 million streamlines, reduced to 2 million using the spherical deconvolution informed filtering of tractograms (SIFT) algorithm [29]. Given that DWI data from the participants in this study were acquired using three different scanners, data harmonisation was performed using ComBat [30, 31], which relies on the software package ‘neuroCombat’ [32]. All processing was performed on SPARTAN, a high‐performance computing cluster [33].

### 2.7 Eighth Nerve Apparent Fibre Density Extraction

**FIGURE 2:**
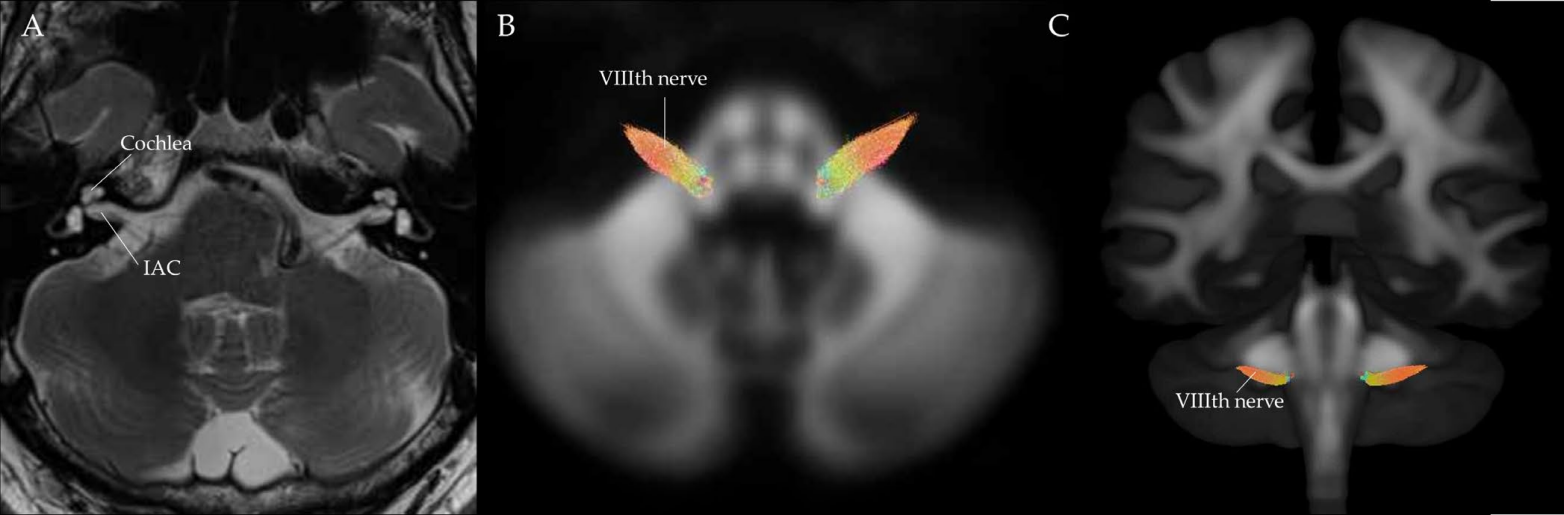
(A) T2‐weighted MR image showing an axial slice acquired from a participant. The cochlea and internal auditory canal (IAC) are clearly identifiable. (B) White matter fibre orientation distribution function population tempate, axial slice, with the VIIIth nerves shown. The VIIIth nerves were generated with probabilitic tractography using the dMRI data. (C) Coronal view of the same image shown in (B). The position of the VIIIth nerves are shown.

The VIIIth nerve was delineated on the white matter study‐specific unbiased ODF population template image using a manual process. This involved demarcating two regions of interest: the end of the VIIIth nerve and the cochlear nucleus within the brainstem. A narrow boundary (exclusion area) was also defined to ensure extraneous tracts were not included. Probabilistic tractography (iFOD2) was then used to generate the VIIIth nerve tracts (shown in Figure 2) between these two regions. The VIIIth nerve tract was subsequently converted into a fixel‐mask for AFD extraction for each individual and statistical analysis to be performed. Different anatomical information obtained from structural (T2‐weighted) MRI and dMRI is shown in Figure 2.

### 2.8 Statistical Analysis

Statistical analyses were performed using Minitab version 19 for Windows. Standard statistical methods were employed to compare the groups within this study. A significance level of *p* < 0.05 was used for all tests unless otherwise specified.

## 3 Results

### 3.1 Group Comparison of Apparent Fibre Densities of the VIIIth Nerve

**FIGURE 3:**
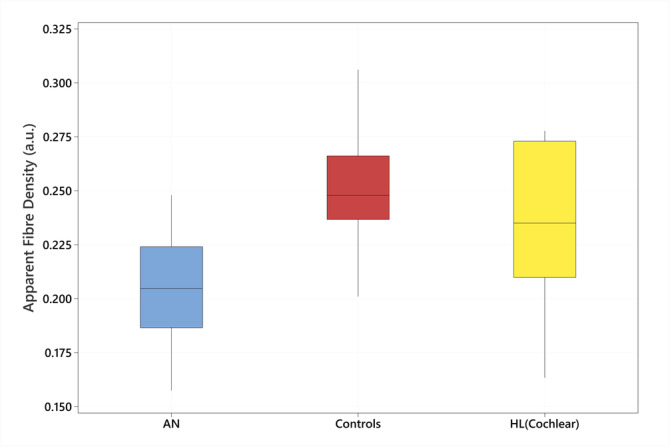
Boxplots showing a comparison of the apparent fibre density (AFD) results obtained from the VIIIth cranial nerve between the groups included in this study: The auditory neuropathy (AN) group, the normal hearing control group (controls), and the cochlear hearing loss group (HL(Cochlear)). The AFD metric is provided AFD values are represented in arbitrary units (a.u.) due to the relative nature of the metric. The boxplots show the median (centre line), the interquartile range (shaded area), and whiskers (range) of the data.

Vestibulocochlear nerve fibre densities varied across participant groups. A one‐way analysis of variance (ANOVA) was conducted to compare the AFD of the VIIIth nerve across the three groups included in this study: Normal hearing controls, AN group and the cochlear hearing loss group. The ANOVA revealed a statistically significant difference in VIIIth nerve AFD results across the three groups, *F*(2,51) = 18.16, *p* < 0.001. The means and standard deviations for each group were as follows: Normal hearing controls (mean = 0.25267, SD = 0.22), AN group (mean = 0.20502, SD = 0.03) and cochlear hearing loss group (mean = 0.23360, SD = 0.04; Figure 3). Post hoc comparisons using the Tukey Honestly Significant Difference test indicated that the mean VIIIth nerve AFD score for the AN group was significantly lower than that of the normal hearing control group (*p* < 0.001) and the cochlear hearing loss group (*p* = 0.04). In comparison, the AFD results for the cochlear hearing loss group did not differ significantly from the controls (*p* = 0.20; Figure 3).

### 3.2 Individual *Z*‐Score Analysis of the Apparent Fibre Densities of the VIIIth Nerve

A total of, 9 of the 18 participants with auditory neuropathy had VIIIth nerve AFD values that were more than 2 SD below the mean established for the normal hearing cohort. In comparison, AFD results were within 2 SD of the mean for all but one participant with cochlear hearing loss. Table 2 presents the individual AFD results for participants with AN and cochlear hearing loss, along with the associated *Z*‐scores generated from comparisons with the normal hearing control participants.

**TABLE 2:** VIIIth nerve apparent fibre density (AFD) extracted from individuals with auditory neuropathy (AN) and cochlear hearing loss (HL).

Subject	VIII nerve AFD	*Z*‐score
AN1	0.21386	−1.8
AN2	0.24801	−0.2
AN3	0.19890	**−2.5**
AN4	0.23914	−0.6
AN5	0.21482	−1.7
AN6	0.17747	**−3.4**
AN7	0.19826	**−2.5**
AN8	0.19519	**−2.6**
AN9	0.22339	−1.3
AN10	0.18932	**−2.9**
AN11	0.21054	−1.9
AN12	0.22638	−1.2
AN13	0.17347	**−3.6**
AN14	0.15745	**−4.4**
AN15	0.19281	**−2.7**
AN16	0.17841	**−3.4**
AN17	0.23911	−0.6
AN18	0.21388	−1.8
HL1	0.27300	0.9
HL2	0.23505	−0.8
HL3	0.25571	0.1
HL4	0.27778	1.2
HL5	0.20992	−1.9
HL6	0.22028	−1.4
HL7	0.16637	**−3.9**

*Note:* *Z*‐scores generated by comparing AFD results against participants with normal hearing control. Statistically significant *Z*‐scores are shown in bold and the *p*‐value is included.

### 3.3 Relationship Between VIIIth Nerve Axonal Density and Sound Detection

**FIGURE 4:**
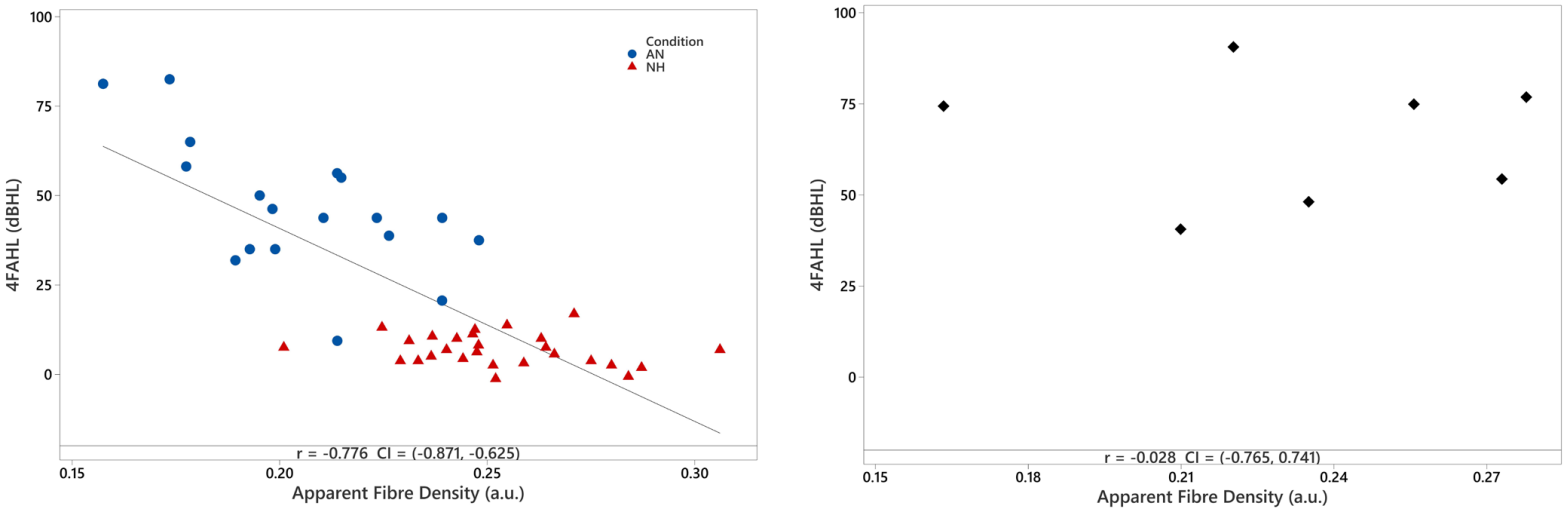
Left panel: Scatterplot showing the relationship between VIIIth nerve apparent fibre density [arbitratry units (a.u.)] results and 4‐frequency average hearing level (dBHL) for Group 1 (participants with ‘normal’ cochlear function). Group 1 is comprised of participants with normal hearing (NH) and auditory neuropathy (AN). Right panel: Scatterplot showing the same relationship for Group 2 (participants with hearing loss resulting from cochlear dysfunction).

To investigate the relationship between axonal density within the VIIIth nerve and pure tone hearing thresholds, study participants were separated into two groups: Group 1 consisted of participants with no evidence of cochlear dysfunction. This included participants with normal hearing and participants with AN who had extant OAEs and/or the presence of a cochlear microphonic (see Table 1 for details). Group 2 consisted of participants with evidence of cochlear pathology (i.e., hearing loss with absent OAEs). Pearson's correlation analyses were performed to examine the relationship between VIIIth nerve AFD and 4‐frequency average hearing thresholds (averaged across both ears) for each participant group. Group 1 results indicated a significant negative correlation between VIIIth nerve AFD and 4FAHL (*r* = −0.776, *p* < 0.001) suggesting that lower VIIIth nerve AFD is associated with poorer sound detection ability (Figure 4, left panel). For Group 2, there was no correlation between AFD and hearing thresholds (*r* = −0.028, *p* > 0.05; Figure 4, right panel).

## 4 Discussion

The findings of this study provide significant insights into the microstructural changes associated with auditory (axonal) neuropathies within the auditory system, especially in relation to the vestibulocochlear (VIIIth) nerve. The observed differences in AFD between participants with AN, cochlear hearing loss and normal hearing controls emphasise the utility of dMRI in detecting and quantifying axonal degeneration within the auditory system.

Additionally, the findings demonstrate that participants with diseases associated with axonopathy exhibit significantly lower VIIIth nerve AFD compared to both individuals with cochlear hearing loss and normal hearing. This reduction in AFD indicates that axonal loss or degeneration within the VIIIth nerve is a prominent feature of axonal neuropathies, which aligns with previous histopathological findings indicating substantial axonal damage in many of these conditions [34, 35, 36, 37, 38]. Importantly, these findings in conjunction with published evidence for specific patient populations [18, 21, 39] further validate the use of AFD as a sensitive marker for assessing the extent of white matter damage within the vestibulocochlear nerve, offering a potential non‐invasive in vivo tool for early diagnosis and monitoring of disease progression.

The majority of our hearing‐impaired participants fell neatly into one of two clinical categories presenting with either ‘sensory’ or ‘neural’ sites of lesion. The 18 patients with genetically confirmed neurodegenerative disease all showed the AN result pattern with evidence of normal cochlear function (extant otoacoustic emissions and/or cochlear microphonics), but absent or abnormal auditory neural responses. Half of these (9/18) subsequently showed significantly reduced auditory fibre densities consistent with vestibulocochlear nerve differentiation. In contrast, six of the seven participants in the cochlear hearing loss group fit the classic sensory pathology pattern with abnormal responses from the cochlear hair cells, auditory evoked neural responses consistent with the degree of hearing loss and normal auditory fibre densities.

One individual (HL7), however, presented with evidence of both cochlear‐ and neural lesion sites. It is uncertain whether there was a causal relationship, but there is evidence from post‐mortem investigations [40, 41] that auditory deprivation (resulting from sensory hearing loss) can be associated with axonal loss—particularly in cases such as this where the hearing loss is of profound degree (right ear) and of prolonged duration. Arguing against the deprivation effect is the fact that the fibre density value for the left side (where the severity of hearing loss was of only mild‐to‐moderate degree) was relatively similar to the right (L: 0.173630; R: 0.159118). The low fibre densities on both sides suggest some other mechanism. Significant noise exposure (as experienced by HL7) has been linked (in animal models) to dendritic damage which causes the terminal dendrites to swell and withdraw from the synaptic connections to the cochlear inner hair cells and subsequent loss of ganglion cells over time [6]. A similar mechanism has been proposed in noise‐exposed humans with ABR findings consistent with dendritic site‐of‐lesion [42]. Additionally, while the complete set of factors influencing the degree and rate of secondary degeneration of spiral ganglion neurons in humans is not known, degeneration is more severe when both the inner hair cells and outer hair cells have been decimated (which is the case with excessive noise exposure) [16], and/or when there is damage to the peripheral terminal processes of cochlear neurons [17]. Another possibility is that the idiopathic sudden hearing loss experienced by HL7 adversely impacted neuronal cell numbers or was associated with some form of neural mechanism. For instance, immune‐mediated inflammation and associated tissue damage caused by the body's response to a virus within the cochlea and auditory system could contribute to both the sensory and neural deficits observed. This is supported by post‐mortem histopathological studies, which indicate both cochlear sensory hair cell damage and neuronal loss is commonplace among individuals with sudden hearing loss [43, 44]. Another consideration is that HL7 was also the oldest participant in this study. Age‐related changes within the auditory system include the loss of both outer and inner hair cells, as well as the degeneration of spiral ganglion cells and axonal fibres [3, 13, 14, 16]. These changes typically begin to show in around the 5th decade of life and accelerate with increasing age [3, 14, 45].

Sound detection thresholds in both adults and children with auditory neuropathies are evenly distributed across the audiometric range, with ≈10% of individuals showing normal hearing levels and a similar proportion presenting as anacusic [22, 46]. The mechanism(s) underlying impaired sound detection are not well understood [47]. This is primarily a reflection of the fact that the integrity of the auditory neural anatomy has not been measurable in vivo (i.e., while patients are still able to provide behavioural hearing levels). This study is the first to explore the relationship between hearing thresholds and the fine‐structure of the auditory nerve in extant participants and suggests that approximately 25% of the ‘typical’ fibre population is necessary to support normal detection. Furthermore, the findings suggest that loss of fibre density below that level is strongly correlated with the degree of hearing loss. Comparatively, in cases where sound detection thresholds are elevated (primarily) due to cochlear hair cell loss, the auditory nerve fibre population is not a good predictor of hearing thresholds. This finding is supported by histopathology studies of cochlear hearing loss, showing that outer and inner hair cell survival, not auditory nerve fibres, is the most accurate predictor of thresholds [6, 48].

Prediction of hearing threshold levels in babies diagnosed with auditory neuropathy at a young age represents a significant clinical challenge. In neurologically normal neonates, sound detection levels are most commonly estimated from ABR threshold (the softest stimulus presentation level that can elicit a repeatable electrophysiologic response [49]) and auditory interventions such as hearing aid fittings can be undertaken in the first weeks of life. For babies with auditory neuropathy (who show absent ABRs at maximum presentation levels) prediction of hearing acuity is not possible, and intervention is typically delayed until the child can provide reliable behavioural responses to sound (≥ 6 months corrected age). As such, an objective technique such as dMRI that can predict hearing thresholds (at least in patients where axonopathy is the primary pathologic process) may form a basis for early intervention and afford the possibility of access to auditory input through critical auditory developmental periods [50].

The study has a number of limitations. Participants were included across a wide age range, which may have influenced the results, as auditory nerve fibres are known to degenerate with age [45]. Hence, the potential confounding effects of age‐related neural degeneration cannot be excluded. However, a proportion of the participants with AN whose VIIIth AFD results fell outside the normative range (Table 2) were relatively young. Additionally, the group with typical cochlear hearing loss was underrepresented (*n* = 7), limiting the statistical power and robustness of correlation analyses within this subgroup. This weakens the ability to draw definitive conclusions about the relationship between VIIIth nerve AFD and hearing thresholds in individuals with cochlear hearing loss.

In summary, this study highlights the utility of dMRI in detecting microstructural changes within the auditory system—specifically the vestibulocochlear nerve. The findings underscore the potential of AFD as a sensitive and non‐invasive marker for axonal degeneration, which may be particularly relevant in cases of auditory (axonal) neuropathy. The distinct differences in AFD among participants with AN, cochlear hearing loss and normal hearing not only validate the clinical relevance of these measures but also open new avenues for early diagnosis and intervention. Furthermore, the strong correlation between AFD and hearing thresholds in cases of AN suggests that dMRI could aid in predicting hearing levels, particularly in challenging clinical scenarios. Additionally, the findings demonstrate the potential multifactorial nature of hearing loss and the need for comprehensive assessments that consider sensory, neural and other contributing factors. Overall, this research lays the groundwork for more targeted, personalised and timely interventions in auditory disorders, especially in vulnerable populations such as infants with auditory neuropathy, where early detection and treatment are critical for optimal auditory development. Future larger‐scale studies are warranted to further validate these findings and explore the potential of dMRI as a clinical tool in the comprehensive assessment and management of hearing impairment.

## Author Contributions

**Julien Zanin:** conceptualization, methodology, investigation, writing – original draft, writing – review and editing, project administration, data curation, formal analysis, resources. **Gary Rance:** conceptualization, methodology, investigation, funding acquisition, writing – review and editing, project administration, resources.

## Ethics Statement

The study was conducted in accordance with the Declaration of Helsinki, and approved by the Human and Research Ethics Committee of the Royal Victorian Eye and Ear Hospital (22‐1525H) and the Institutional Review Board of the Ethics Committee of PLAGH (S2018‐042‐01).

## Consent

Informed consent was obtained from all subjects involved in the study.

## Conflicts of Interest

The authors declare no conflicts of interest.

## Data Availability

The data that support the findings of this study are available on request from the corresponding author. The data are not publicly available due to privacy or ethical restrictions.
